# Poikiloderma with neutropenia, Clericuzio-type: Case report of a 7-year-old Syrian child

**DOI:** 10.1097/MD.0000000000047046

**Published:** 2026-01-16

**Authors:** Ammer Alabed, Maha Al haj Kadour, Nour Alshaar, Aisha Alloush, Basheer Khalil

**Affiliations:** a Faculty of Medicine, University of Damascus, Damascus, Syria; b Department of Pediatrics, Children’s University Hospital, Faculty of Medicine, University of Damascus, Damascus, Syria.

**Keywords:** case report, chronic neutropenia, Clericuzio-type: *USB1* gene mutations, poikiloderma with neutropenia, recurrent infections

## Abstract

**Introduction:**

Poikiloderma with neutropenia (PN), also known as Clericuzio-type, is a rare autosomal recessive disorder characterized by immune system defects. This condition manifests in infancy with a distinctive rash that spreads over time, accompanied by various systemic symptoms, including recurrent infections and chronic neutropenia. Understanding this disorder is crucial for timely diagnosis and management, especially given its significant morbidity and potential mortality.

**Case presentation:**

We present a case of a 7-year-old Syrian male diagnosed with PN. He presented to the pediatric department with right flank pain, a triphasic fever pattern, dysuria, and dysphagia. His medical history included recurrent respiratory infections and a hyperpigmented rash since infancy. Clinical examination revealed significant symptoms, including developmental delays, pallor, and abnormal nails.

**Clinical discussion:**

The case emphasizes the clinical challenges in diagnosing PN, particularly given overlapping features with other inherited poikilodermas. Characteristic symptoms include the presence of poikiloderma, neutropenia, and recurrent infections. The autosomal recessive inheritance pattern noted in this case aligns with the documented familial patterns in existing literature. Clinicians must maintain a high index of suspicion and consider a comprehensive evaluation in patients exhibiting similar clinical signs to ensure accurate diagnosis and effective management.

**Conclusion:**

This Syrian case of PN illustrates how a carefully reasoned clinical diagnosis, in the absence of genetic testing, can guide effective care in low-resource contexts. It highlights pragmatic pathways (infection surveillance, antimicrobial escalation, and preventive counseling) that remain actionable even when confirmatory genetics are unavailable.

## 1. Introduction

Poikiloderma with neutropenia (PN), often referred to as Clericuzio-type, is a rare autosomal recessive immune system defect.^[1]^ The condition initially appears during infancy as a red, raised rash on the limbs. Over time, the rash gradually spreads inward toward the body. As it fades, it leaves behind areas of both lighter and darker pigmentation.^[2]^ Short stature, facial dysmorphisms, and nail abnormalities are clinical features often observed in PN. Additional manifestations may include recurrent infections, chronic neutropenia, hepatosplenomegaly, and various abnormal laboratory findings.^[1,3]^ The manifestation of the defect may encompass various dermal conditions, including poikiloderma, hyperkeratotic nails, and palmoplantar hyperkeratosis.^[3]^ The various homozygous or compound heterozygous mutations in the U6 Biogenesis 1 (*USB1*) gene have been implicated in the development of the PN, Clericuzio-type.^[4]^ This condition was 1st documented in 1991 by Clericuzio among Navajo Native Americans, manifests cutaneously and hematologically.^[5]^ Mortality of PN patients is significantly increased by cancer and recurrent infections. Despite a significant decline in infection frequency beyond the 1st decade of life, bronchiectasis, productive cough, and reactive airway disease persist throughout adulthood.^[3]^

We present a case of PN, Clericuzio-type in a 7-year-old Syrian male.

## 2. Case presentation

### 2.1. Presentation

**Figure 1.**
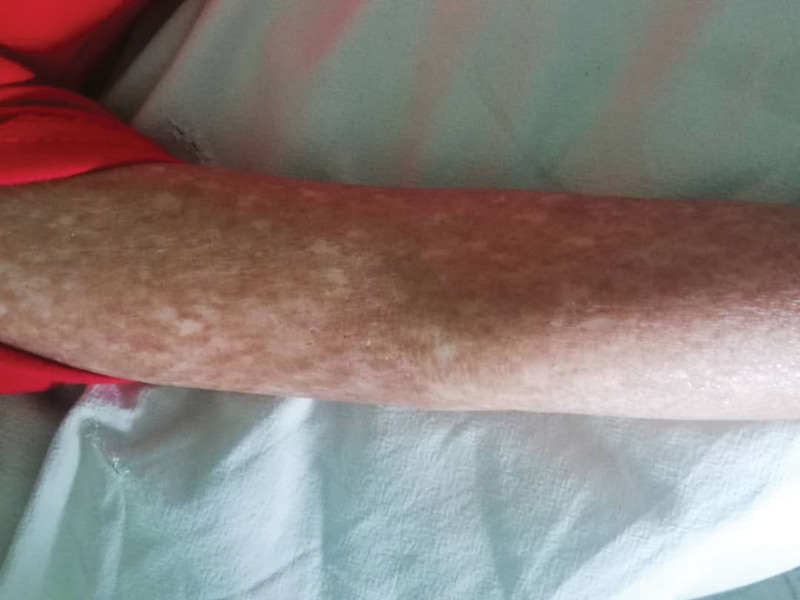
Poikiloderma with mottled pigmentation.

**Figure 2.**
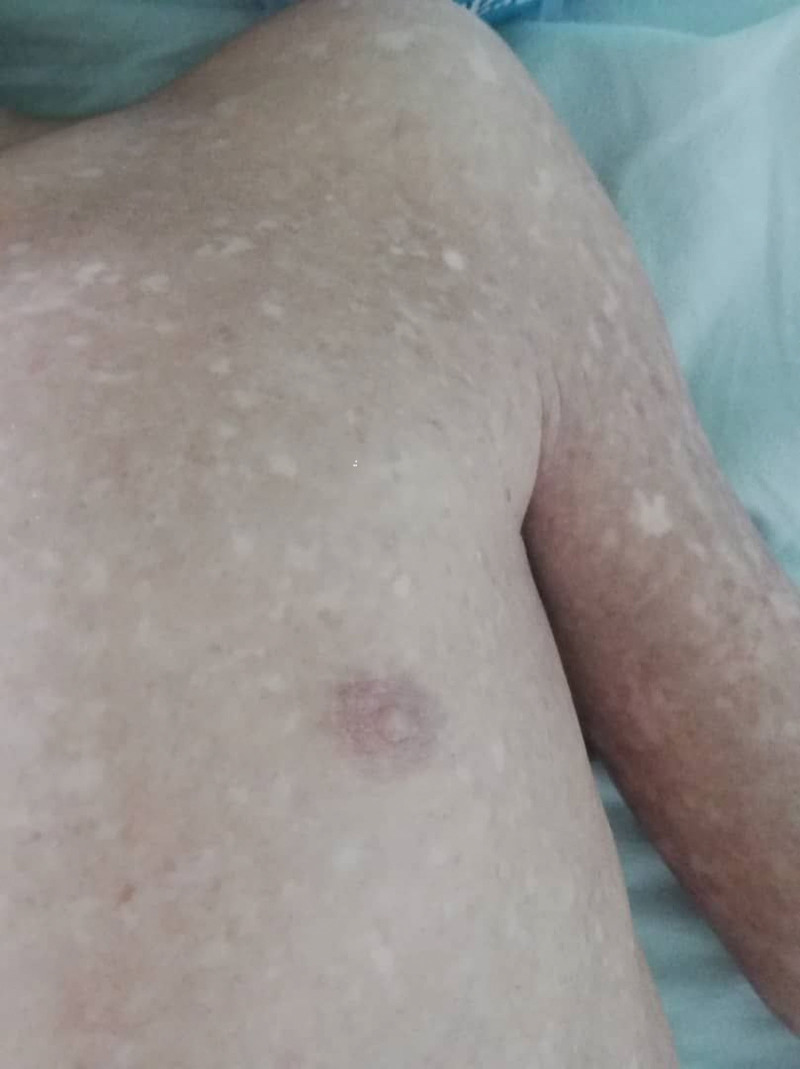
Sparse, light-colored scalp hair reflecting ectodermal involvement.

**Figure 3.**
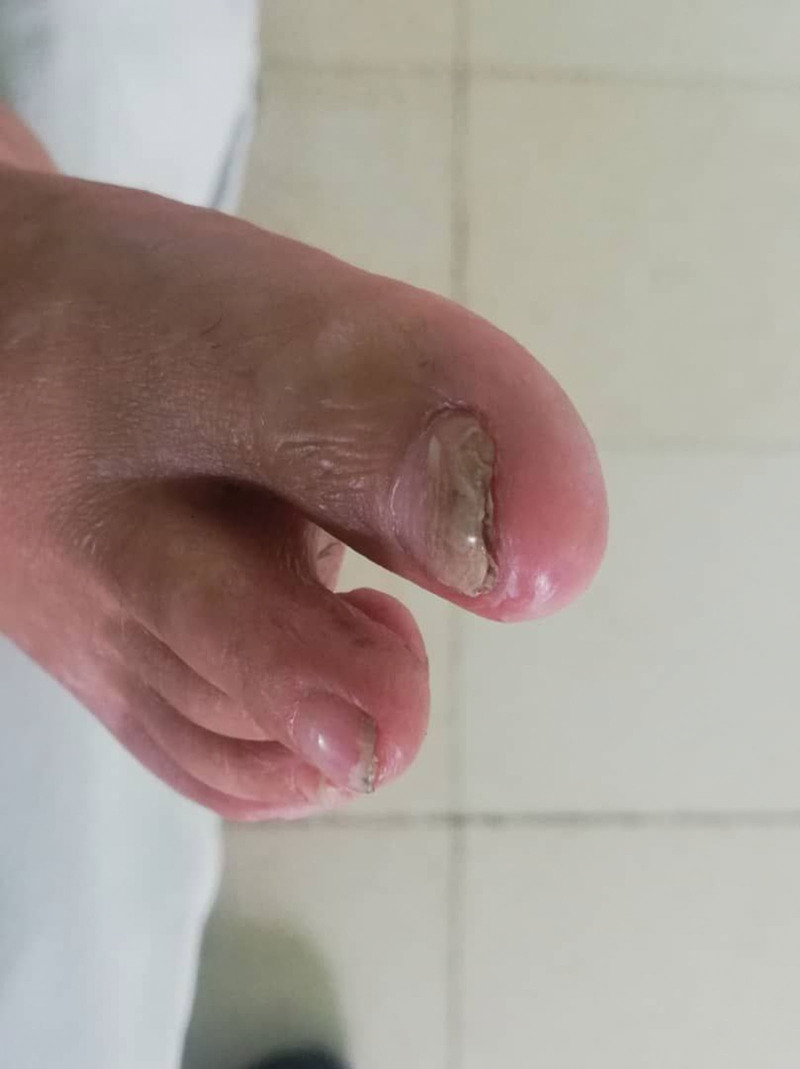
Thickened, dystrophic nails consistent with pachyonychia.

**Figure 4.**
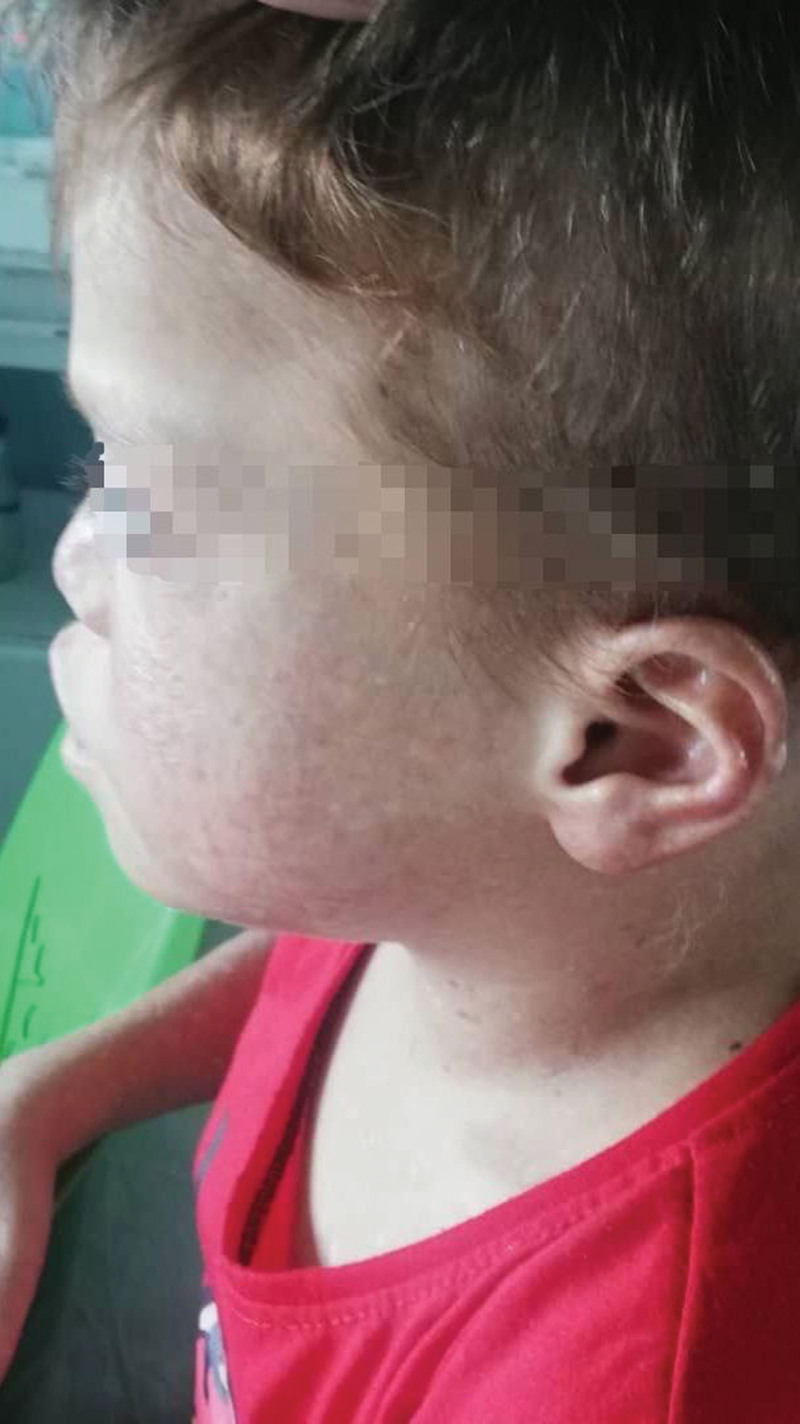
Distinctive craniofacial features.

A 7-year-old male patient presented to the pediatric department with right flank pain and a triphasic fever pattern, reaching 39°C. During he experienced dysuria and dysphagia, medical history included recurrent respiratory infection and hyperpigmented rash consistent with skin melanosis since the age of 1 month (Fig. 1). Furthermore, the patient exhibited developmental delay, characterized by a lack of interaction with the environment, fear of strangers, and delayed speech development (limited to 1-word utterances at the age of 2 years). Family history is significant for a hyperpigmented rash consistent with skin melanosis in his brother’s and niece’s son. The patient was 18 kg and 107-cm tall. Clinical examination revealed blond appearance, paleness, thinness (Fig. 2), restricted finger movement, abnormal nails (Fig. 3), lenticular lymphadenopathy in the anterior neck triangle, an increased anteroposterior chest diameter, a 3/6 systolic murmur, and palpable right flank fullness attributed to hepatomegaly and splenomegaly. We recognized a small genitalia size in addition to heightened tendon reflexes, saddle nose, low ear set (Fig. 4), and multiple dental caries.

### 2.2. Work up

Laboratory tests were conducted immediately upon administration: white blood cells = 6400 cells/µL, neutrophils = 49%, hemoglobin = 5 g/dL, mean corpuscular volume = 64 fL, platelets = 9000 cells/µL, lactate dehydrogenase = 864 U/L, uric acid = 4 mg/dL, and phosphorus = 3.6 mg/dL.

Two days later, a bone marrow biopsy showed a normocellular marrow with megaloblastic changes and erythroid maturation arrest, consistent with ineffective erythropoiesis likely due to vitamin B9/B12 deficiency, in the context of the underlying PN.

The patient was diagnosed with PN clinically, considering the distribution of skin lesions since birth, the elevated lactate dehydrogenase, chronic neutropenia, and bone marrow morphology consistent with ineffective hematopoiesis. Molecular genetic testing for the detection of biallelic *USB1* pathogenic variants was not performed due to financial constraints.

### 2.3. Treatment

The patient was initially managed with intravenous antibiotics targeting respiratory and urinary tract infections. During hospitalization for the renal abscess, he received piperacillin and tazobactam at 112.5 mg/kg/d for 21 days, in combination with amikacin at 7.5 mg/kg/d for 7 days.

After 7 days of this initial regimen, levofloxacin was introduced at 10 mg/kg/d for 14 days. Additional short courses included sulbactam for 2 days at 30,000 U/kg/d, and colistin for 21 days at 30,000 U/kg/d, which was initiated to control inflammation and prevent recurrent infection.

Supportive therapy included vitamin B12 supplementation at 50 µg intramuscularly once weekly and folic acid at 1 mg orally once daily.

### 2.4. Patient perspective

The patient’s parents expressed deep concern over their child’s recurrent infections and persistent skin changes, which began during infancy. They described the long diagnostic journey as stressful but were relieved to finally receive a clear clinical explanation for his condition. They reported good adherence to sun protection and vaccination recommendations and expressed appreciation for the multidisciplinary care provided.

### 2.5. Follow-up

Preventive measures include annual influenza vaccination to reduce respiratory infection risks and the liberal application of sunscreens to minimize the likelihood of skin cancer. Surveillance protocols involve yearly medical evaluations by a physician familiar with PN, including complete blood count, dermatological screenings for skin cancer beginning at the age of 10.

## 3. Discussion

This report provides a comprehensive, clinically anchored characterization of PN in a child from Syria. It is an uncommon genetic skin condition that results from biallelic mutations in the *USB1* gene and is marked by early-onset poikiloderma alongside persistent neutropenia.^[4]^ Mostefai et al documented a family from Morocco with 3 siblings presenting with characteristic features of PN, reinforcing the autosomal recessive inheritance pattern. Similarly, Van Hove et al,^[6,7]^ the inheritance of the condition was believed to follow an autosomal recessive pattern, as 8 out of the 1st 14 documented cases were siblings, while neither parent exhibited symptoms.^[2]^ The primary challenge for any clinician is to diagnose this condition based on their current symptoms and signs. Even though the core diagnostic features include poikiloderma, neutropenia, recurrent infections, and nail abnormalities that remain consistent across cases, this task becomes more difficult when multiple diseases share similar characteristics. A clear example of this is found in inherited poikilodermas such as dyskeratosis congenita, PN, and Rothmund–Thomson syndrome (RTS).^[8]^ Overlapping clinical features, especially poikiloderma, complicate the accurate diagnosis and classification of conditions. Table 1 delineates the distinctions among these diseases.^[9–12]^ In our setting, the absence of confirmatory genetic testing limits diagnostic certainty. However, the constellation of clinical features, together with systematic comparison to overlapping syndromes, supports PN as the most likely diagnosis. This highlights the challenge faced in low-income countries, where reliance on clinical reasoning remains essential in the absence of molecular confirmation. This issue is increased in low-income countries due to limited access to genetic testing. Van Hove et al case suggested that previously reported cases of RTS with myelodysplasia and neutropenia might actually represent PN rather than RTS,^[7]^ highlighting the importance of distinguishing between these syndromes.

**Table 1:** Distinguishing features of poikiloderma with neutropenia (PN), Rothmund–Thomson syndrome (RTS), and dyskeratosis congenita (DC).

Feature	Rothmund–Thomsen syndrome (RTS)	Poikiloderma with neutropenia (PN)	Dyskeratosis congentia
Genetics	Autosomal recessive mutations in the RECQL4 gene	Autosomal recessive mutations in the *USB1* gene	X-linked recessive mutations in dyskerin
Main clinical features	Poikiloderma, sparse hair, small stature, cataracts	Poikiloderma, neutropenia, recurrent infections	Nail dysplasia, abnormal skin pigmentation, and oral leukoplakia
Onset	Infancy	Early childhood	Variable
Dermatological signs	Poikiloderma, photosensitivity	Poikiloderma, no photosensitivity	Dysplastic fingernails, reticular skin pigmentation
Bone abnormalities	Radial ray defects, absent/small thumbs	No radial ray defects	Microcephaly, avascular necrosis of the hips and shoulders.
Growth	Short stature	Short stature	Short stature
Immune system	Generally normal, some cases of immunodeficiency	Neutropenia, recurrent infections	Lymphopenia, decreased T-, B-, and natural killer cell count, hypogammaglobulinemia

PN can exhibit a diverse range of symptoms, including hypogonadotropic hypogonadism, enlargement of the liver and spleen, along with other unusual characteristics.^[4]^ Development delay was observed in our patient, whereas other reported literature presents with no history of psychomotor developmental delay.^[13]^ However, Akio Tanaka et al documented motor delay in a PN case,^[14]^ suggesting that neurological involvement may vary among affected individuals.

Chronic neutropenia predisposes patients to an increased risk of recurrent respiratory infections, which may lead to meningitis and ulcers.^[13]^ Akdogan et al reported that severe osteomyelitis has been documented in PN patients, leading to digit loss.^[4]^ This condition has the potential to result in mortality due to complications from infection.^[7]^ These complications emphasize the critical need for close monitoring, early intervention, and multidisciplinary care in PN patients. Additionally, chronic skin thinning, changes in skin pigmentation increase the risk for skin cancers,^[2]^ which also has an impact on the severity of the prognosis and mortality rate.

## 4. Conclusion

This report provides a comprehensive, clinically anchored characterization of PN in a child from Syria. The case documents a serious infectious complication, renal abscess, and the management process. PN is rarely reported from Syria and similar low-resource settings. The case presented here highlights the importance of recognizing the clinical features and familial patterns associated with PN, as early diagnosis and intervention are crucial for managing complications. The necessity for continued research into this condition is underscored by its complex genetic underpinnings, particularly involving mutations in the *USB1* gene. Furthermore, the application of preventive measures, such as annual vaccinations and diligent sun protection, is critical for improving the quality of life and reducing mortality associated with skin cancer in affected individuals. As awareness increases, it is imperative for healthcare professionals to consider PN in differential diagnoses for patients with similar presentations, ensuring timely and appropriate care.

### 4.1. Limitation

Enetic confirmation (*USB1* sequencing) was not performed due to financial constraints, introducing residual diagnostic uncertainty despite a strongly suggestive phenotype (early-onset poikiloderma, chronic neutropenia, recurrent infections). In addition, post-discharge outcomes and adherence could not be verified because the patient did not return for scheduled follow-up. As a single-patient report from a resource-limited setting, findings may not be generalizable.

## Acknowledgments

We would like to express our sincere gratitude to *VISION RESEARCH* and *Medical Research Empowerment* for their valuable assistance and insights during the preparation of this case report. Both have granted permission to be acknowledged by name.

## Author contributions

**Conceptualization:** Ammer Alabed.

**Methodology:** Ammer Alabed.

**Project administration:** Ammer Alabed, Nour Alshaar, Aisha Alloush.

**Resources:** Nour Alshaar, Aisha Alloush.

**Supervision:** Basheer Khalil.

**Validation:** Maha Al haj kadour.

**Visualization:** Maha Al haj kadour, Basheer Khalil.

**Writing – original draft:** Ammer Alabed, Maha Al haj kadour, Nour Alshaar, Aisha Alloush, Basheer Khalil.

**Writing – review & editing:** Ammer Alabed, Maha Al haj kadour, Nour Alshaar, Aisha Alloush, Basheer Khalil.

## References

[1] Roebke LJ Vander Maten JW Alkhoury G. Hyperbaric oxygen management of recurrent cellulitis in poikiloderma with neutropenia. Am J Med Genet A. 2021;185:2150–2. 33836117 10.1002/ajmg.a.62204

[2] McKusick VA. Poikiloderma with neutropenia, Clericuzio-type. 2021. https://www.omim.org/entry/604173. Accessed May 15, 2025.

[3] Piccolo V Russo T Di Pinto D. Poikiloderma with neutropenia and mastocytosis: a case report and a review of dermatological signs. Front Med (Lausanne). 2021;8:680363. 34179048 10.3389/fmed.2021.680363 PMC8222900

[4] Akdogan N Kindis E Bostan E Utine E Alikasifoglu M Ersoy-Evans S. Poikiloderma with neutropenia, Clericuzio-type accompanied by loss of digits due to severe osteomyelitis. J Clin Immunol. 2020;40:934–9. 32620997 10.1007/s10875-020-00815-5

[5] Farruggia P Indaco S Dufour C. Poikiloderma with neutropenia: a case report and review of the literature. J Pediatr Hematol Oncol. 2014;36:297–300. 23823120 10.1097/MPH.0b013e31829f35e7

[6] Mostefai R Morice-Picard F Boralevi F. Poikiloderma with neutropenia, Clericuzio type, in a family from Morocco. Am J Med Genet A. 2008;146A:2762–9. 18925663 10.1002/ajmg.a.32524

[7] Van Hove JL Jaeken J Proesmans M. Clericuzio type poikiloderma with neutropenia is distinct from Rothmund–Thomson syndrome. Am J Med Genet A. 2005;132A:152–8. 15558713 10.1002/ajmg.a.30430

[8] Walne AJ Vulliamy T Beswick R Kirwan M Dokal I. Mutations in C16orf57 and normal-length telomeres unify a subset of patients with dyskeratosis congenita, poikiloderma with neutropenia and Rothmund–Thomson syndrome. Hum Mol Genet. 2010;19:4453–61. 20817924 10.1093/hmg/ddq371 PMC2957322

[9] Savage SA. Dyskeratosis congenita and telomere biology disorders. Hematology Am Soc Hematol Educ Program. 2022;2022:637–48. 36485133 10.1182/hematology.2022000394 PMC9821046

[10] Siitonen HA Kopra O Kääriäinen H. Molecular defect of RAPADILINO syndrome expands the phenotype spectrum of RECQL diseases. Hum Mol Genet. 2003;12:2837–44. 12952869 10.1093/hmg/ddg306

[11] Wang L Clericuzio C Larizza L Concolino D. Poikiloderma with neutropenia. In: Adam MP Feldman J Mirzaa GM Pagon RA Wallace SE Amemiya A, eds. GeneReviews(®). University of Washington; 1993. Seattle Copyright © 1993-2025, University of Washington, Seattle. GeneReviews is a registered trademark of the University of Washington, Seattle. All rights reserved. 1993. 29072891

[12] Wang LL Gannavarapu A Kozinetz CA. Association between osteosarcoma and deleterious mutations in the RECQL4 gene in Rothmund–Thomson syndrome. J Natl Cancer Inst. 2003;95:669–74. 12734318 10.1093/jnci/95.9.669

[13] Chekr J Andraws J Elias J Alasmar D. Poikiloderma with neutropenia: a case report. J Med Case Rep. 2025;19:16. 39810212 10.1186/s13256-025-05027-2 PMC11734469

[14] Tanaka A Morice-Picard F Lacombe D. Identification of a homozygous deletion mutation in C16orf57 in a family with Clericuzio-type poikiloderma with neutropenia. Am J Med Genet A. 2010;152A:1347–8. 20503306 10.1002/ajmg.a.33455

